# Epigenetics of bipolar disorder: a bibliometric landscape and visualization analysis

**DOI:** 10.3389/fpsyt.2026.1804155

**Published:** 2026-06-05

**Authors:** Lan Xiong, Haixia Fan, Jie Gao, Minmin Lei

**Affiliations:** 1 Department of Neurology, The First People’s Hospital of Guangyuan, Guangyuan, China; 2 Department of Sleep Center, First Hospital of Shanxi Medical University, Taiyuan, China; 3 Shanxi Medical University, Taiyuan, China; 4 Zigong Fourth People’s Hospital, Zigong, China

**Keywords:** bibliometric analysis, bipolar disorder, epigenetics, gene regulation, psychiatric disorder

## Abstract

**Background:**

The incidence of bipolar disorder (BD) is an ongoing global public health concern, driving a heightened focus on elucidating its underlying pathophysiology. Mounting evidence indicates that epigenetic mechanisms play a pivotal role in the onset and progression of BD, presenting promising avenues for identifying novel biomarkers and therapeutic targets. However, a comprehensive bibliometric mapping of the intellectual structure and research frontiers in this rapidly evolving field is currently lacking.

**Methods:**

A systematic literature search was conducted to retrieve relevant publications spanning from January 1995 to October 2025, utilizing both the Web of Science Core Collection (WoSCC) and Scopus databases. Following rigorous screening and deduplication processes, bibliometric and visual analyses were executed using VOSviewer, CiteSpace, and the Bibliometrix R package. We systematically evaluated publication trends, collaboration networks (countries, institutions, and authors), and core themes.

**Results:**

A total of 1154 valid documents were included in the final analysis. The United States emerged as the most productive and influential country in this domain, with Harvard University identified as the leading institution. Kato T and Nöthen MM were recognized as the most prolific author and highly co-cited scientist, respectively. Molecular Psychiatry stood out as the core journal, featuring both the highest publication volume and citation frequency. Keyword co-occurrence and burst detection revealed that current research frontiers predominantly encompass histone modifications, HDAC inhibitors, long noncoding RNAs (lncRNAs), and the interplay between genetic variants (e.g., SNPs, CNVs) and epigenetic regulation.

**Conclusion:**

This bibliometric study delineates the developmental trajectory of epigenetic research in BD. The field is actively shifting from fundamental epigenetic mechanisms towards the exploration of dynamic regulatory networks (e.g., lncRNAs and HPA axis) and multi-omics integration. These findings provide researchers with a structural overview of current hotspots and highlight robust directions for future clinical translation and targeted epigenetic interventions.

## 1 Introduction

Bipolar disorder (BD) is a chronic psychiatric condition characterized by alternating manic and depressive episodes. The global burden of this disorder is substantial, with an estimated 12-month prevalence of 1.5% and a lifetime prevalence of 2.4%. Furthermore, the global incidence of BD continues to rise steadily, disproportionately impacting women and adolescents. The clinical severity of BD is underscored by alarmingly high mortality rates, as 6% to 7% of afflicted individuals die by suicide (1, 2). Additionally, patients frequently exhibit persistent deficits across various cognitive domains, even during euthymic periods (3).

While the exact etiology of BD remains elusive, epigenetics plays a crucial role in elucidating its pathogenesis by demonstrating how non-genetic and environmental factors influence gene expression. Epigenetic mechanisms encompass modifications—such as DNA methylation and non-coding RNA regulation—that alter gene activity or cellular characteristics without changing the underlying DNA sequence (4). Recent research has identified significant associations between BD and epigenetic regulatory mechanisms, particularly within neuronal cells where disease-associated signatures primarily target these networks. For instance, studies have highlighted disease-associated regulatory modules involving the allele-specific expression of long non-coding RNAs, which contribute to synaptic and behavioral abnormalities in BD (5, 6).

Beyond broad regulatory networks, specific epigenetic modifications hold significant promise as clinical biomarkers for diagnostics and personalized treatments. Epigenetic alterations in candidate genes such as brain-derived neurotrophic factor (BDNF), Catechol-O-methyltransferase (COMT), and FKBP5 have been strongly associated with the disorder (7). Furthermore, genome-wide studies have identified 191 differentially methylated positions in BD patients compared to healthy controls, indicating accelerated biological aging and a profound epigenetic association with the disease (8).

Accordingly, it is of critical importance to further elucidate the epigenetic mechanisms underlying BD, identify reliable novel biomarkers, and explore innovative therapeutic strategies. However, to the best of our knowledge, a comprehensive bibliometric mapping of this rapidly evolving field remains lacking. Thus, to enable researchers to gain a systematic understanding of the research status and developmental trends, we employed bibliometric approaches to perform a quantitative analysis and visualization of the literature pertaining to BD and epigenetics.

## 2 Methods

### 2.1 Research design and scope

The data were retrieved from the Web of Science Core Collection (WoSCC) and Scopus from 1995 until the date of the search on 18 October 2025. Both databases were utilized to ensure comprehensive coverage and minimize the risk of missing relevant publications. WoSCC is a prominent academic database known for its extensive coverage of scientific and authoritative works, dependable citation indexing, and detailed citation data, including keywords and references, making it the most commonly used resource in bibliometric research (9). Scopus complements WoSCC by indexing a broader range of journals, offering extensive coverage in the social sciences and life sciences, and providing robust author and affiliation level metrics that facilitate cross-disciplinary citation analyses and trend detection.

### 2.2 Search strategy and inclusion criteria

To formulate a comprehensive search query, we consulted the Medical Subject Headings (MeSH) database and related entry terms sourced from PubMed. Crucially, these MeSH terms were used strictly as a reference to identify relevant synonyms and free-text terms, which were subsequently applied to the search strings in WoSCC and Scopus. The keywords “Bipolar Disorder” and “epigenetic” were utilized with Boolean logic and appropriate parentheses grouping to construct precise search strings for each specific database. The exact search strategies were developed as follows:WoSCC: ((TI=(“bipolar disorder*”) OR AB=(“bipolar disorder*”) OR AK=(“bipolar disorder*”)) AND (TI=(“epigenetic*”) OR AB=(“epigenetic*”) OR AK=(“epigenetic*”)))Scopus: (TITLE-ABS-KEY(“bipolar disorder*”) AND TITLE-ABS-KEY(“epigenetic*”)). The search strategy focused on identifying publications published between January 1, 1995, and October 18, 2025. The inclusion criteria were limited to original articles written in English. To ensure data quality, non-original research documents such as reviews, meeting abstracts, editorials, and letters were excluded. Raw data were first curated in R to screen out and remove duplicate records across the two databases, a critical preprocessing step to guarantee dataset accuracy and uniqueness.

### 2.3 Data processing and parameters

Scopus data were first converted into a plain text format compatible with the Web of Science (WOS) using CiteSpace (6.4.R1). Raw data were then curated in R (4.5.1) to remove duplicates. Bibliometric analyses and descriptive statistics were conducted using Bibliometrix (4.4.3) and Excel to summarize major countries/regions, journals, authors, bublication trends and distributions.

**Figure 1:**
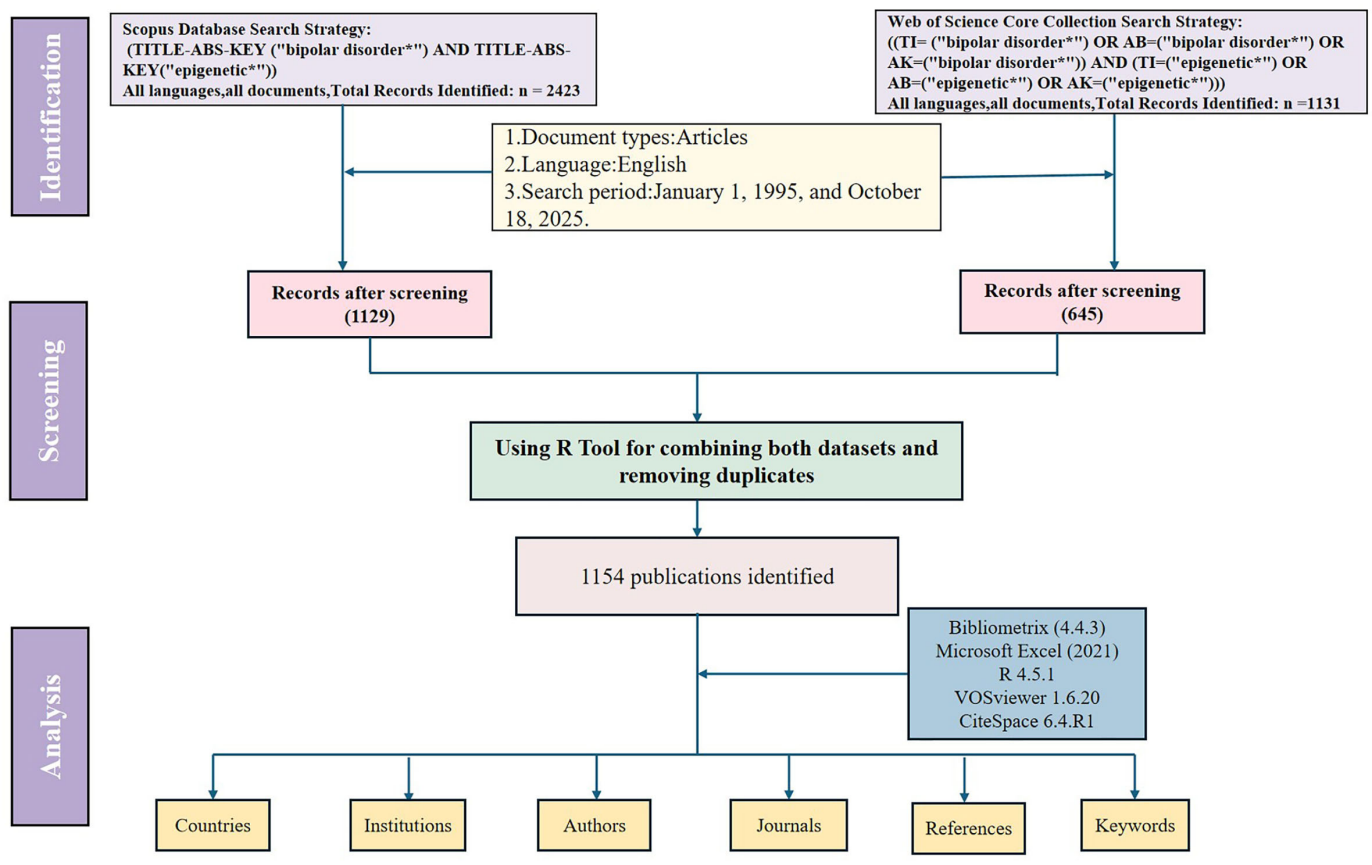
The flowchart for literature search, selection and analysis. TI, title; AK, author keywords; AB, abstract.

Bibliometric network analyses were performed with VOSviewer (1.6.20) and CiteSpace (6.4.R1). In VOSviewer, full counting was applied, with thresholds set as follows: sources = 5, keywords = 5, co-organizations = 6, co-countries = 12, co-authors = 4, co-cited authors = 24, co-cited sources = 72. CiteSpace was used for temporal mapping, clustering, and burst detection, with time slices from 2005 to 2025 (1 year per slice) and a g-index (k = 25). Network pruning was conducted using Pathfinder and pruning sliced networks. When discrepancies occurred, Bibliometrix results were prioritized (**Figure 1**).

## 3 Results

### 3.1 Annual publications trends and distributions of countries/regions

**Figure 2:**
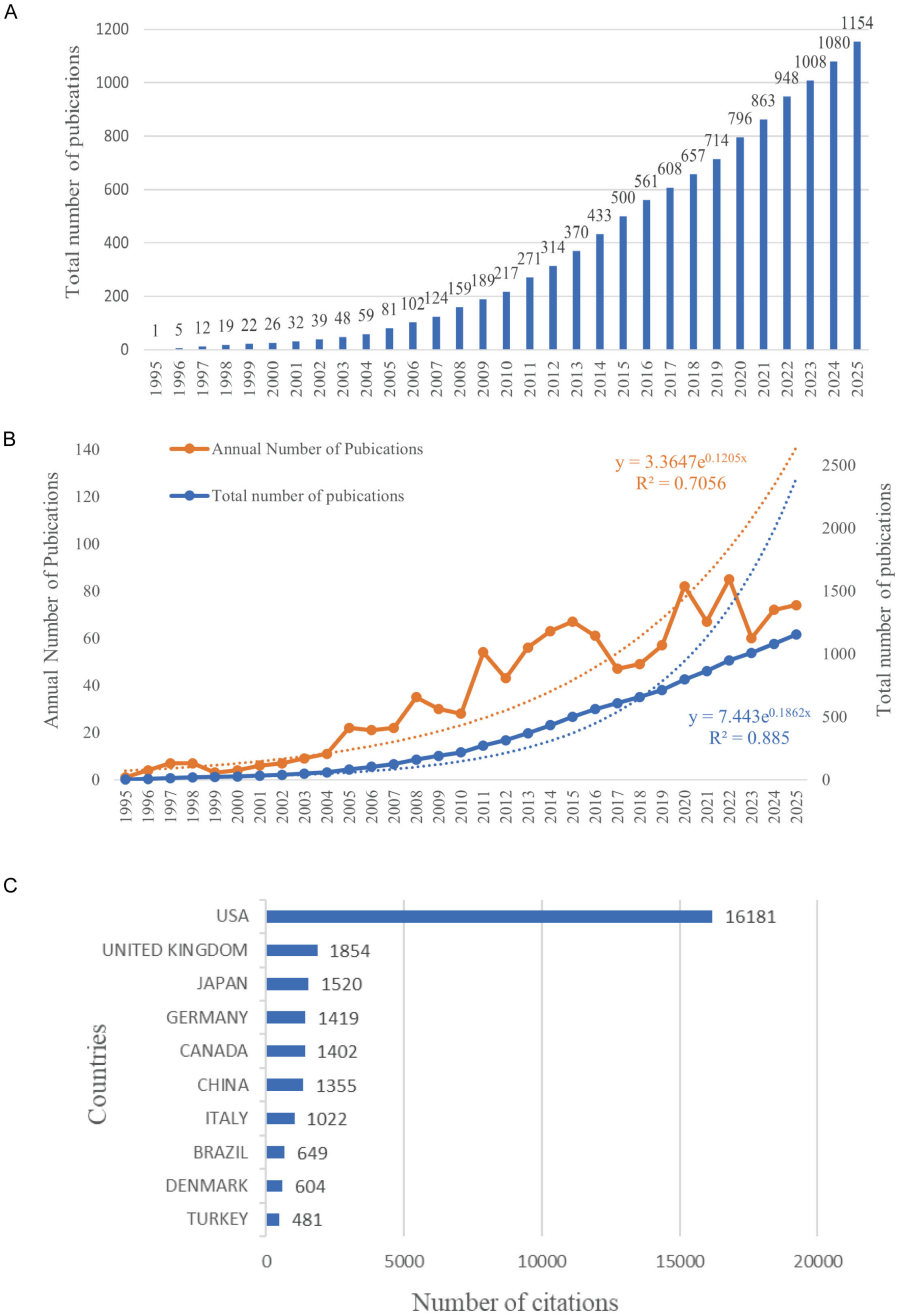
Publication trends and distributions of countries/regions. **(A)** Total Publication Volume; **(B)** Price’s Law of Cumulative Growth Curve Fitting Analysis; **(C)** Top 10 Countries/Regions by Citation Impact.

A total of 1, 154 publications spanning from 1995 to 2025 were included in the final analysis, providing a comprehensive overview of research development in this field over the past three decades. As illustrated in **Figure 2A**, the cumulative number of publications has grown steadily, achieving an annual growth rate of 15.43% (**Supplementary Table 4**). Based on the annual publication dynamics and exponential trend modeling (R^2^ = 0.885 for cumulative growth) (**Figure 2B**), the temporal distribution can be rationally delineated into three distinct phases. The initial exploration phase (1995–2005) was characterized by slow and fluctuating growth with limited outputs. The second phase (2006–2010) marked a period of steady growth, reflecting an accumulating academic interest in the field. Notably, the third phase (2011–2025) demonstrates a rapid, exponential expansion. The publication output during the past fifteen years constitutes the vast majority of the total dataset, a surge that closely coincides with the increasing recognition of epigenetics in the diagnostic and therapeutic relevance of bipolar disorder.

**Figure 3:**
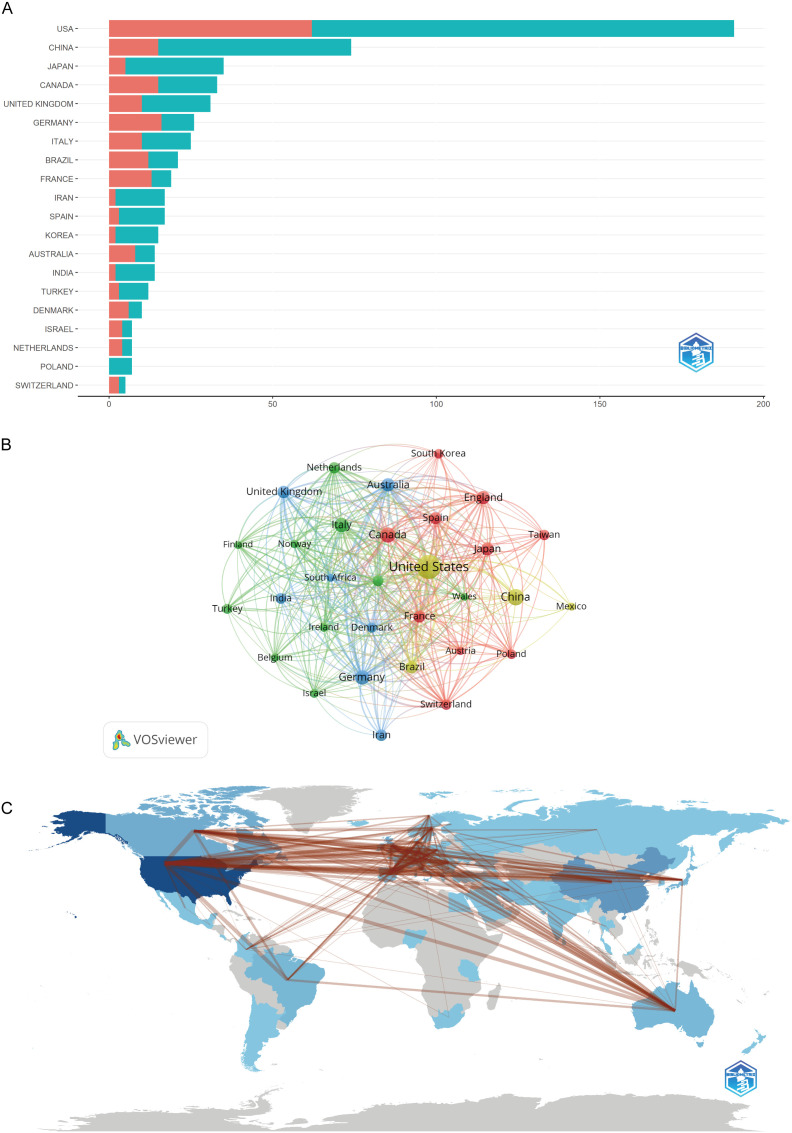
Country/regional publication volume and cooperation patterns. **(A)** the top 10 countries responsible for the number of studies (SCP: Single Country Publications, indicating that the authors of the article are from the same country; MCP: Multiple Country Publications, indicating that the author of the article is from multiple countries, that is, there is cooperation between countries). **(B)** the Country/Region cooperation network based on the VOSviewer (each point represents a Country/Region, the size of the node represents the number of publications, and the thickness of the lines between nodes represents the intensity of cooperation between Country/Region). **(C)** Country/Region Collaboration Map. Darker shades of blue indicate higher collaboration rates, and the width of the link lines represents the strength of collaboration between two countries.

Geographic and country-level analyses highlight a highly globalized, yet distinctly centralized, research landscape. The United States exerts a dominant influence in this domain, ranking overwhelmingly first in both total publications (**Figure 3A**) and total citations (16, 181) (**Figure 2C**), alongside a previously noted high average citation rate per article. The United Kingdom, Japan, and Germany closely follow in terms of academic impact. Notably, China has emerged as the second-largest contributor in absolute document output, establishing itself and Japan as major research hubs in the Asian region (**Supplementary Table 5**).

Further analysis of Single-Country Publications (SCP) and Multiple-Country Publications (MCP) reveals diverse collaborative strategies among leading nations (**Figure 3A**). While the United States leads in the absolute volume of internationally co-authored articles (MCP), China’s robust output remains predominantly domestically driven, evidenced by a high proportion of SCP. Conversely, European nations—particularly France, Germany, and Italy—demonstrate strong international integration, with France exhibiting a notably high MCP ratio (68.4%) (**Supplementary Table 5**). These collaborative dynamics are further corroborated by network and geographic visualizations (**Figures 3B, C**), which visually identify the United States as the central global nexus, bridging extensive academic connections across North America, Europe, and East Asia.

### 3.2 Distributions of institutions and authors

**Figure 4:**
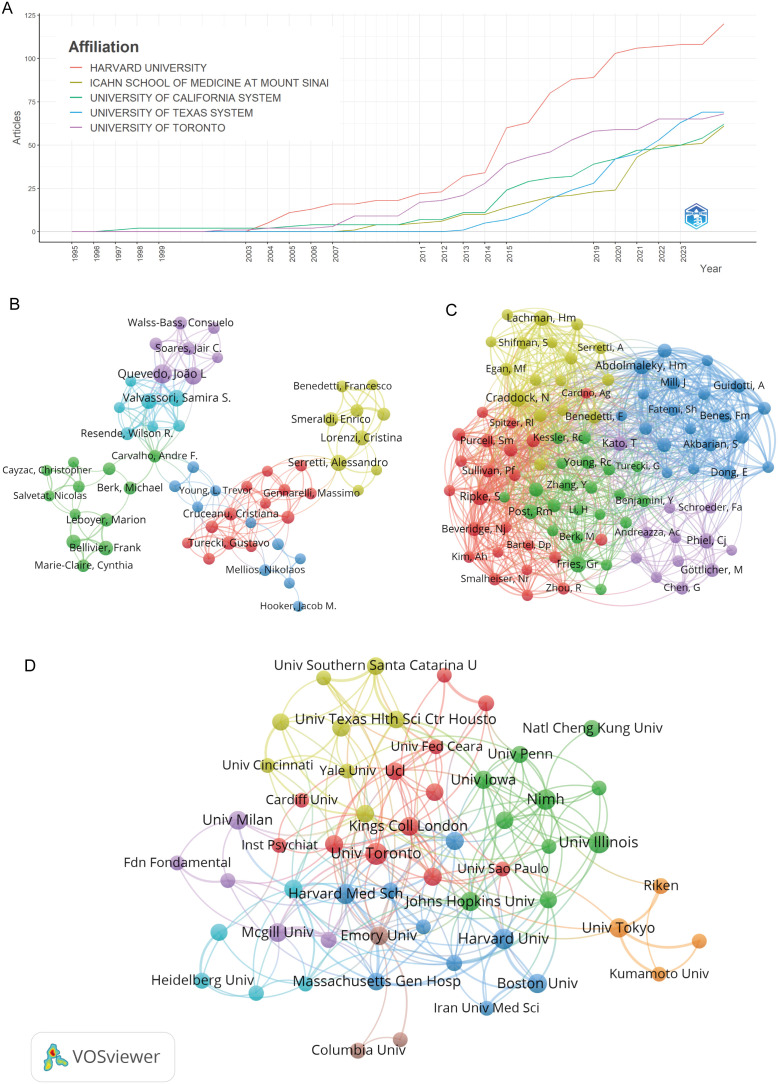
Visualization and analysis of institutional involvement and author. **(A)** Annual Publication Counts of the Top 5 Institutions. **(B)** Co-authorship Authors network based on VOSviewer. **(C)** Co-Cited Author Clustering Analysis based on VOSviewer. Each circle represents an author, with lines indicating collaborative ties. The different colors of the circles denote collaborative clusters among authors. Node sizes in this network reflect the frequency of co-citations. **(D)** the Institutions cooperation network based on the VOSviewer (each point represents an Institution, the size of the node represents the number of publications, and the thickness of the lines between nodes represents the intensity of cooperation between Country/Region).

As detailed in **Supplementary Table 6**, Harvard University, the University of Texas System, and the University of Toronto represent the most prolific institutions in this field, contributing 120, 69, and 68 articles, respectively. The temporal dynamics of publication output among the top affiliations (**Figure 4A**) reveal that systematic research in this domain was virtually nonexistent prior to 2000. Output experienced a sustained increase starting around 2003, transitioning into a rapid developmental phase post-2014. Harvard University maintains an absolute leadership position, characterized by a long-term, continuous, and systematic research advantage. The University of Toronto closely follows, demonstrating consistent scholarly output and significant international influence. Conversely, while the University of Texas System entered the field later (showing prominent activity post-2013), it exhibited a steep, high-growth trajectory after 2018, categorizing it as a latecomer with rapidly expanding academic influence. Furthermore, the institutional collaboration network (**Figure 4D**) visualizes the global cooperative infrastructure. It highlights the University of Toronto, Harvard University, and the National Institute of Mental Health (NIMH) as central hubs, represented by dense clusters (e.g., red, blue, and green nodes) that facilitate robust research partnerships. This network spans globally, with notable contributions from other major entities such as the University of Tokyo and Stanford University.

A total of 7, 220 authors have contributed to publications in this field. Highlighting the highly collaborative nature of this research, only 47 authors have published single-author works. Furthermore, the average number of co-authors per document is 9.66, with 18.37% of publications involving international teams (**Supplementary Table 4**). **Supplementary Table 1** summarizes the top 10 most impactful authors, ranked by their h-index. T. Kato ranks first (h-index = 16, 25 articles), followed closely by J. Quevedo (h-index = 15, 21 articles). The co-authorship network (**Figure 4B**) maps the collaborative structure of the field, identifying distinct research clusters and demonstrating the interdisciplinary alliances that drive the domain (such as the prominent collaborative cluster led by J. Quevedo and S.S. Valvassori).

In terms of academic impact, M.M. Nöthen achieved the highest total citations (4, 230 TC) among the top authors. Notably, Nöthen’s fractionalized article count is exceptionally low (0.36) relative to their 16 total publications (**Supplementary Table 1**). This bibliometric signature indicates that their massive citation impact is primarily derived from participation in highly cited, large-scale multi-author consortia (such as landmark genome-wide association studies) rather than individual publications. Finally, Analysis of author collaborations uncovered five distinct research clusters (**Figure 4C**), each with its own unique collaborative characteristics.With Ripke, S and Purcell, SM as the core—recognized as the most influential scholar in the red cluster—their organization, the Psychiatric Genomics Consortium (PGC), proposed that psychiatric disorders share a polygenic risk framework, with cross-diagnostic risk loci successfully identified in their studies (10, 11). Led by Merikangas, KR, the green cluster focuses on the epidemiology and clinical management of bipolar spectrum disorders. Supported by data and guidelines from major initiatives and organizations such as the National Comorbidity Survey Replication, the International Society for Bipolar Disorders (ISBD), and the Canadian Network for Mood and Anxiety Treatments (CANMAT), this cluster stresses the importance of phenotypic complexity when interpreting epigenetic findings (12, 13). Represented by Abdolmaleky, HM and Mill, J, the blue cluster highlights epigenetic modifications and transcriptional regulation as critical drivers of pathogenesis, and has identified hypomethylation of the MB-COMT promoter as a key risk marker (14, 15). The yellow cluster, under the leadership of Craddock, N—a pivotal hub in the field—also relied heavily on the collaborative data of the Psychiatric Genomics Consortium (PGC). They demonstrated that complex psychiatric disorders stem from the cumulative effect of numerous small-effect single-nucleotide polymorphisms (SNPs) (16) and revealed substantial genetic overlap among five major psychiatric disorders (17). The purple cluster suggests the revelation of the epigenetic mechanism by which drugs such as sodium valproate exert their therapeutic effects by inhibiting histone deacetylase (HDAC) (18) and confirms the key role of mitochondrial gene expression abnormalities and dysfunction in the pathological process of bipolar disorder (19).

Additionally, network topology analysis identified Ripke, S and Craddock, N—both core figures within the PGC—as central hubs that connect different research domains.

### 3.3 Journals and co-journals

The top 15 most prolific journals published a combined 294 articles, accounting for 25.47% of the total document output (**Supplementary Table 2**). Molecular Psychiatry emerged as the leading journal across multiple metrics, boasting the highest impact factor (IF = 10.1, Q1), the largest publication volume (42 articles), and the highest total citation count (3, 300 citations). According to the Journal Citation Reports (JCR), the majority of these leading journals are classified within the Q1 or Q2 quartiles, reflecting the rigorous academic standard of this field. Notably, while the Proceedings of the National Academy of Sciences of the United States of America (PNAS) published only nine articles, it accumulated 2, 152 citations. This disproportionately high citation rate ranks it second overall in total citations, underscoring its pivotal role in disseminating highly influential, milestone studies. Other journals demonstrating substantial citation impact include Translational Psychiatry (1, 734 citations) and Schizophrenia Research (1, 596 citations).

**Figure 5:**
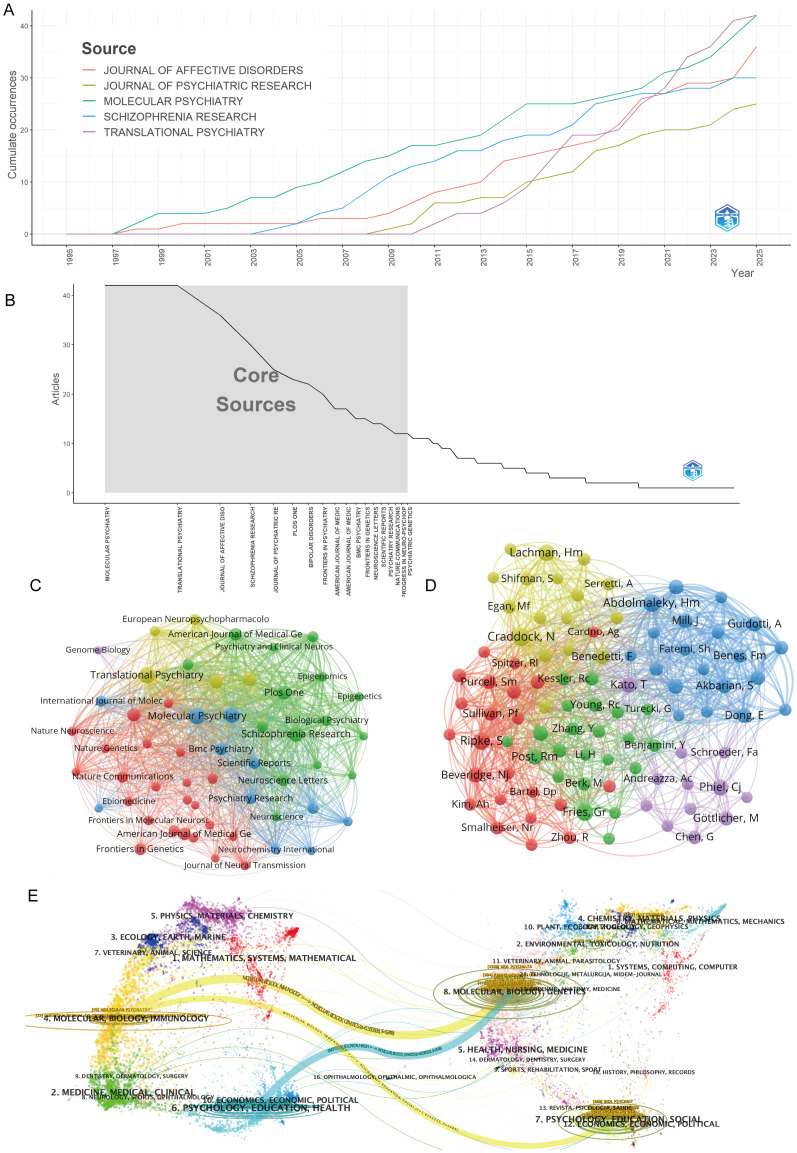
Visualization of journal association networks. **(A)** Annual Publication Counts of the Top 5 Journals. **(B)** Bradford’s law analysis based on the bibliometrix package. **(C)** Co-Cited Journals Clustering Analysis based on VOSviewer. **(D)** Cited Journals network based on VOSviewer. Each circle represents a Journal, with lines indicating collaborative ties. The different colors of the circles denote collaborative clusters among Journals. Node sizes in this network reflect the frequency of co-citations. **(E)** Journal Dual-Map Overlay Analysis: Dual-map visualization showing citing and cited journal relationships, with lines indicating citation flows andknowledge transfer.

The cumulative publication trends among the top journals (**Figure 5A**) illustrate a continuous and dynamic expansion of the literature. Molecular Psychiatry demonstrated a sustained upward trajectory from 1997 to 2015, experienced a brief plateau between 2015 and 2017, and subsequently resumed robust growth with a marked acceleration post-2021. Translational Psychiatry, despite its later inception, exhibited the steepest exponential growth curve. This rapid ascent reflects its strong alignment with contemporary, high-priority research directions. Overall, the marked acceleration in journal output post-2015 suggests that the epigenetic mechanisms of bipolar disorder have crystallized as a major research hotspot, likely propelled by parallel advances in genome-wide association studies and related molecular technologies.

Bradford’s law of scattering (**Figure 5B**) revealed a highly skewed distribution of journal output, wherein a small subset of core journals contributes a disproportionate share of the literature. The identified “core sources” zone is prominently anchored by Molecular Psychiatry, Translational Psychiatry, and the Journal of Affective Disorders, which serve as the primary platforms for scholarly communication and major advances in mental health research. Consistent with this bibliometric law, both temporal trends and scattering patterns validate Molecular Psychiatry as the foundational pillar of this research area.

Journal co-citation networks (**Figures 5C, D**) mapped the intellectual structure of the field, identifying distinct but closely connected research communities. Central hubs within this network—most notably Molecular Psychiatry, Biological Psychiatry, and PNAS—exert profound influence, bridging disparate clusters of psychiatric and biological research. Furthermore, the dual-map overlay (**Figure 5E**) visualizes the interdisciplinary citation pathways and macro-level knowledge flows. The citing journals (representing the research fronts) are predominantly located in the “Molecular, Biology, Immunology” and “Psychology, Education, Health” regions, while the cited references (the intellectual base) are heavily concentrated in the “Molecular, Biology, Genetics” disciplines. The prominent, thick citation trajectories confirm that clinical psychiatric and psychological research relies heavily on foundational discoveries from molecular biology and genetics journals. This underscores the deep interdisciplinary integration required to advance the understanding of epigenetics in bipolar disorder.

### 3.4 References and articles

**Supplementary Table 3** lists the top 10 most heavily cited publications in this field, which constitute its core knowledge base. Among them, the meta-analysis by Lohmueller et al. is the most cited (1, 672 citations) (20), establishing the foundational theory that multiple common variants with weak effects collectively contribute to complex disease susceptibility, thereby emphasizing the necessity of large-scale cohort studies. Subsequent large-scale genome-wide association studies (GWAS) (10, 21), have further revealed the shared genetic mechanisms among psychiatric disorders. For instance, a schizophrenia GWAS identified cross-diagnostic susceptibility loci, including *MIR137* and *CACNA1C*, highlighting the role of gene regulatory networks in cross-disorder mechanisms (10), Another GWAS covering 30 loci emphasized the critical roles of ion channels and synaptic components, providing robust evidence for biologically distinguishing bipolar I from bipolar II disorder through genetic correlation analysis (21).

**Figure 6:**
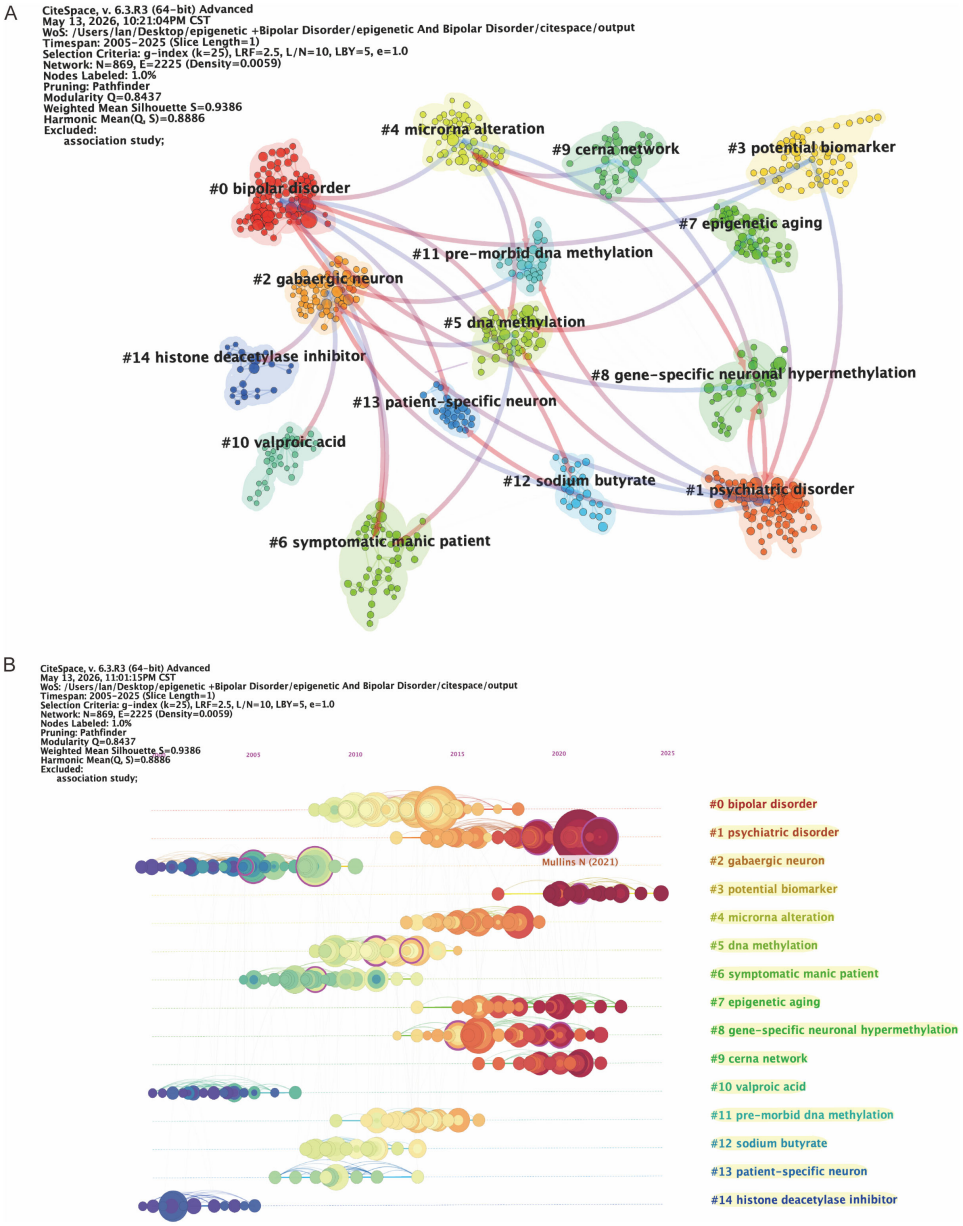
Analysis of references. **(A)** Co-cited references Clustering Analysis based on CiteSpace. **(B)** Co-citation reference clustering timeline produced by CiteSpace.

Literature co-citation clustering analysis based on CiteSpace (**Figure 6A**) revealed the primary research themes in this field. The network demonstrated high modularity (Q = 0.8437) and a high mean silhouette score (S = 0.9386), indicating a significant and highly reliable clustering structure. The major clusters encompass not only macroscopic disease classifications such as “bipolar disorder (#0)” and “psychiatric disorder (#1), “ but also delve into specific epigenetic mechanisms like “DNA methylation (#5), “ “gene-specific neuronal hypermethylation (#8), “ and “histone deacetylase inhibitors (#14)”.

The timeline visualization (**Figure 6B**) delineates the evolutionary trajectory of epigenetic research in bipolar disorder from 2005 to 2025, revealing a series of overlapping and progressively evolving phases rather than discrete transitions. Early studies primarily focused on core disease characterization and fundamental neurobiological and epigenetic mechanisms, including DNA methylation, GABAergic dysfunction, and pharmacological modulation via agents such as valproic acid and histone deacetylase inhibitors (#0, #2, #5, #10, #14). This was followed by a translational phase characterized by exploratory biomarker identification and closer linkage to clinical phenotypes, including microRNA alterations and studies involving symptomatic manic patients (#3, #4, #6). More recent research reflects a shift toward technology-driven mechanistic expansion and multi-omics integration, encompassing emerging themes such as epigenetic aging, gene-specific neuronal hypermethylation, ceRNA regulatory networks, premorbid epigenetic alterations, and patient-specific neuronal models (#7–#9, #11–#13). This temporal evolution reflects the field’s paradigm shift from macroscopic disease characterization toward molecular-level mechanism dissection, individualized neuronal modeling, and exploration of emerging regulatory networks.

**Figure 7:**
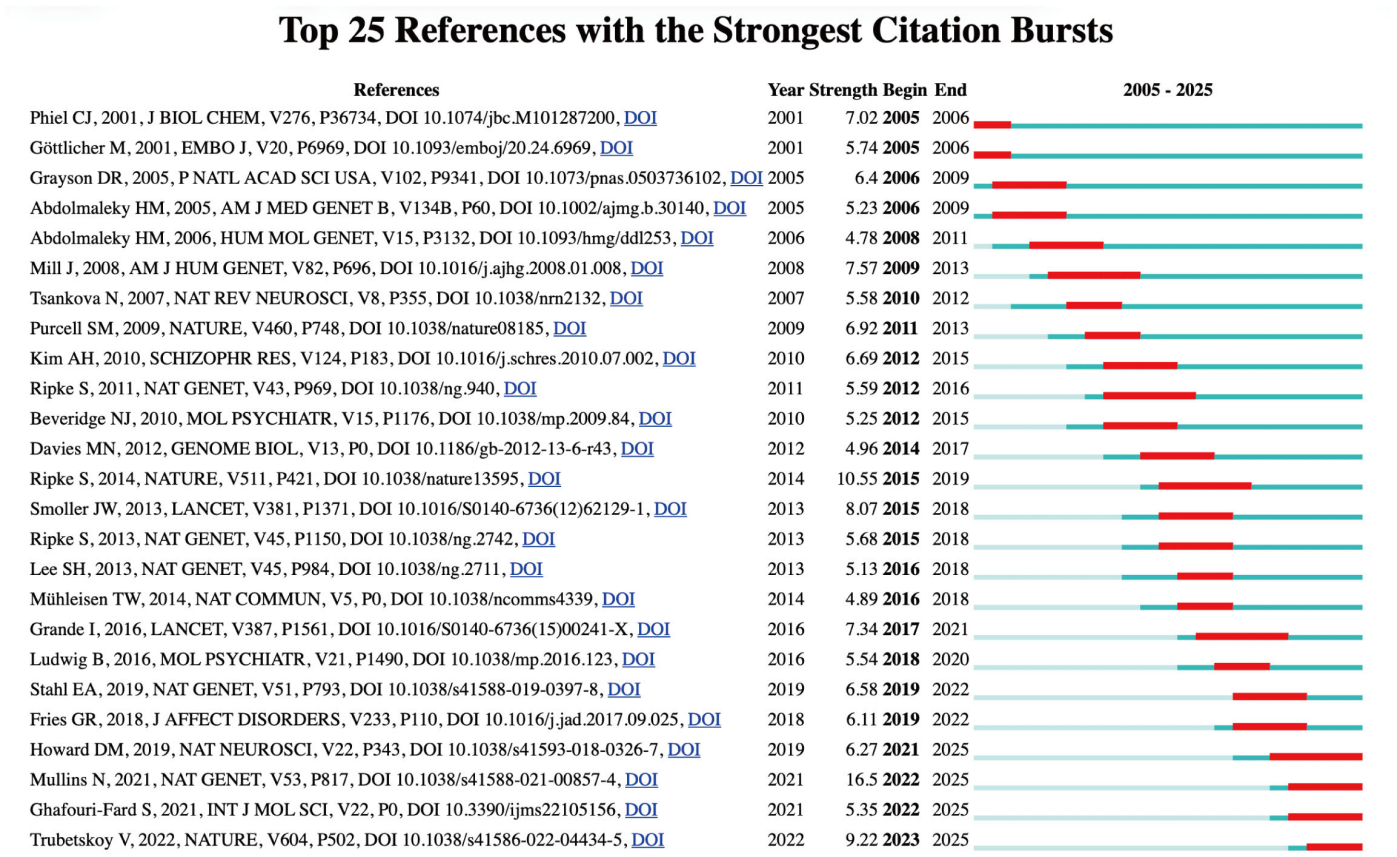
Top 25 references. Top 25 references with the strongest citation bursts. The strongest citation bursts time period was indicated in red.

Literature burst analysis (**Figure 7**) displays key publications with sudden surges in citation frequency between 2005 and 2025, serving as a reliable indicator for identifying research frontiers. In recent years, studies with high burst strengths have primarily focused on exploring the shared genetic architecture of psychiatric disorders. For example, the study by Mullins N (2021) exhibited a remarkable burst strength of 16.5 in 2022 (22). emphasizing the enrichment of synaptic signaling genes and drug targets (e.g., *HTR6*, *FURIN*) in bipolar disorder. Meanwhile, Trubetskoy V (2022, burst strength 9.22) (23). These high-impact studies provide critical insights into shared genetic architecture, therapeutic targets, and cross-disorder mechanisms between bipolar disorder and schizophrenia. reported an overlap between schizophrenia-associated loci and neurodevelopmental genes. These recent high-impact publications indicate that cross-disorder epigenetic mechanisms and the discovery of potential therapeutic targets represent the core research hotspots for both the present and the foreseeable future.

### 3.5 Keyword analysis

**Figure 8:**
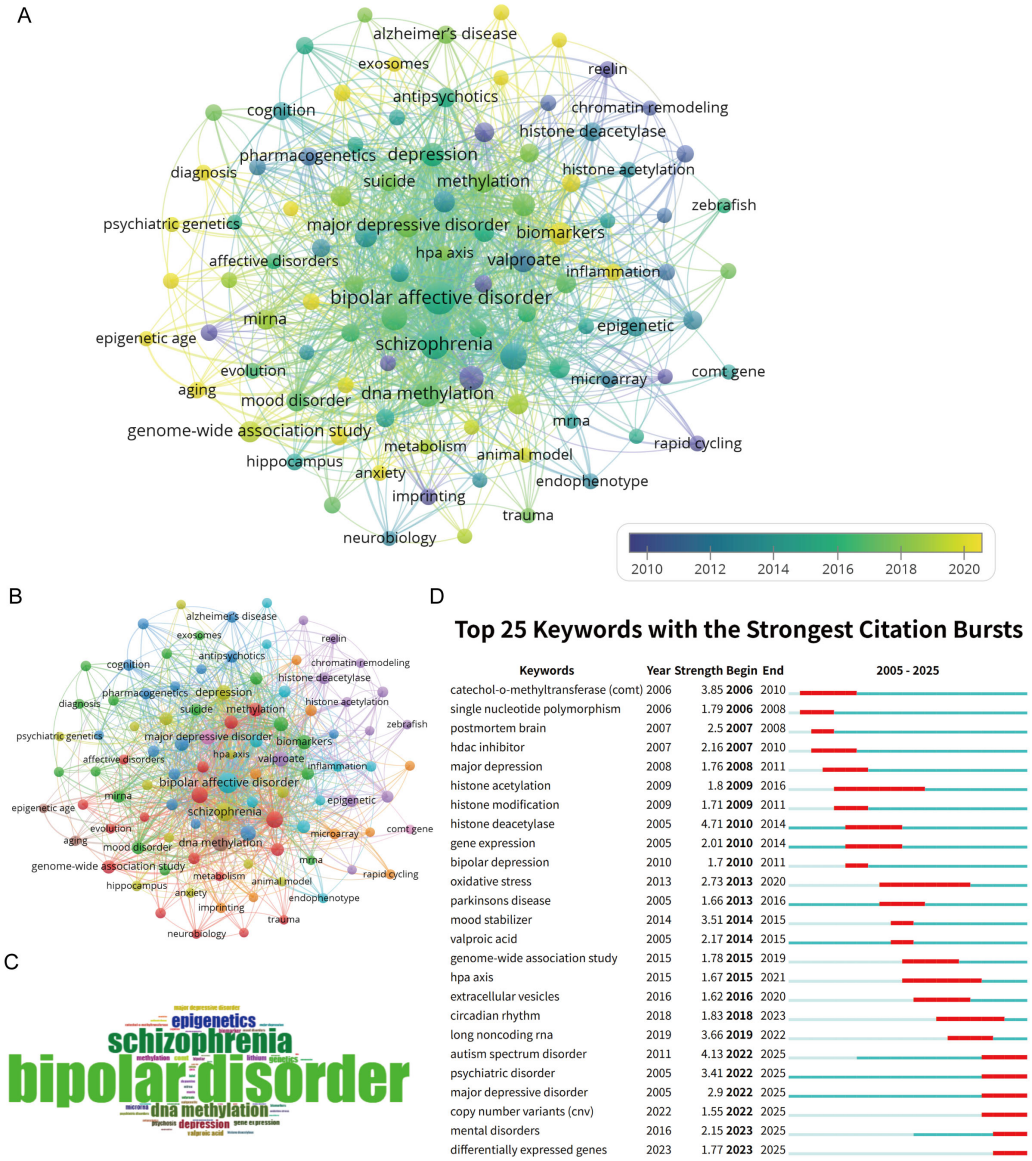
Visualization of keywords. **(A)** Timeline Visualization of Author keywords Collaborations (Node size represents the publication volume of each institution, line thickness indicates the strength of collaborative ties, and node colors distinguish between different collaborative clusters). **(B)** Co-occurrence Author keywords Clustering Analysis based on VOSviewer. **(C)** keywords word cloud based on bibliometrix package. **(D)** CiteSpace visualization map of the top 25 keywords with the strongest citation bursts.

Keyword analysis serves as a robust indicator of the fundamental themes and focal points within a specific research domain. A total of 8, 704 keywords, comprising 2, 476 author keywords and standardized KeyWords Plus, were extracted to map Epigenetic Studies in BD (**Supplementary Table 4**). The word cloud (**Figure 8C**) visually highlights the most prominent research subjects, with “Bipolar Disorder, “ “Schizophrenia, “ “Epigenetics, “ and “DNA Methylation” emerging as the core nodes. This finding aligns with the VOSviewer network visualization (**Figure 8B**), which illustrates tightly interconnected clusters centered around psychiatric diagnoses, genomic variations, and drug therapies. Notably, epigenetics bridges the gap between genetic predisposition and phenotypic expression, with DNA methylation being the most extensively studied mechanism. Furthermore, the prominent display of “Lithium” and “Valproic Acid” underscores the field’s sustained focus on elucidating the pharmacological mechanisms of first-line mood stabilizers.

To trace the evolutionary trajectory of these research themes, an overlay visualization was generated (**Figure 8A**), utilizing a color gradient from purple/blue (earlier publications) to yellow (recent publications). The temporal distribution reveals a distinct paradigm shift in research priorities. Keywords predominantly in blue, such as valproate, histone deacetylase, and reelin, indicate early research hotspots (approximately 2010–2014) that laid the foundational understanding of epigenetic drug targets. Themes subsequently transitioned to macro-level genetic architectures, represented by green nodes (2014–2018) like schizophrenia, DNA methylation, and genomics. Most recently, the emergence of yellow nodes, including biomarkers, transcription factors, GWAS, and machine learning, indicates that the current research landscape is pivoting toward precision medicine, advanced computational algorithms, and the identification of robust clinical markers.

Keyword burst analysis (**Figure 8D**) identifies topics that have garnered intense, surging academic attention over specific periods, providing critical insights into emerging frontiers. The top 25 keywords with the strongest citation bursts can be systematically categorized into five primary domains: (1) Genetics and genomics (e.g., single nucleotide polymorphism, genome-wide association study); (2) Epigenetic regulation (e.g., histone deacetylase, HDAC inhibitor, valproic acid, long noncoding RNA); (3) Neuropsychiatric diagnoses (e.g., major depressive disorder, autism spectrum disorder); (4) Systemic physiological mechanisms (e.g., oxidative stress, HPA axis, circadian rhythm); and (5) Molecular functions and pathways (e.g., gene expression, extracellular vesicles, differentially expressed genes).

Crucially, the burst patterns since 2020 highlight a sustained and growing interest in the epigenetic regulation of BD. Keywords such as autism spectrum disorder, psychiatric disorder, copy number variants (CNV), and differentially expressed genes have exhibited strong bursts extending through 2025. This enduring trend highlights a critical paradigm recognition: understanding the interplay between structural genomic variations (like CNVs) and transcriptomic changes is essential for unraveling the pathophysiology of mental disorders. These active frontiers represent the most promising avenues for the development of novel epigenetic biomarkers and targeted therapeutic interventions.

## 4 Discussion

**Figure 9:**
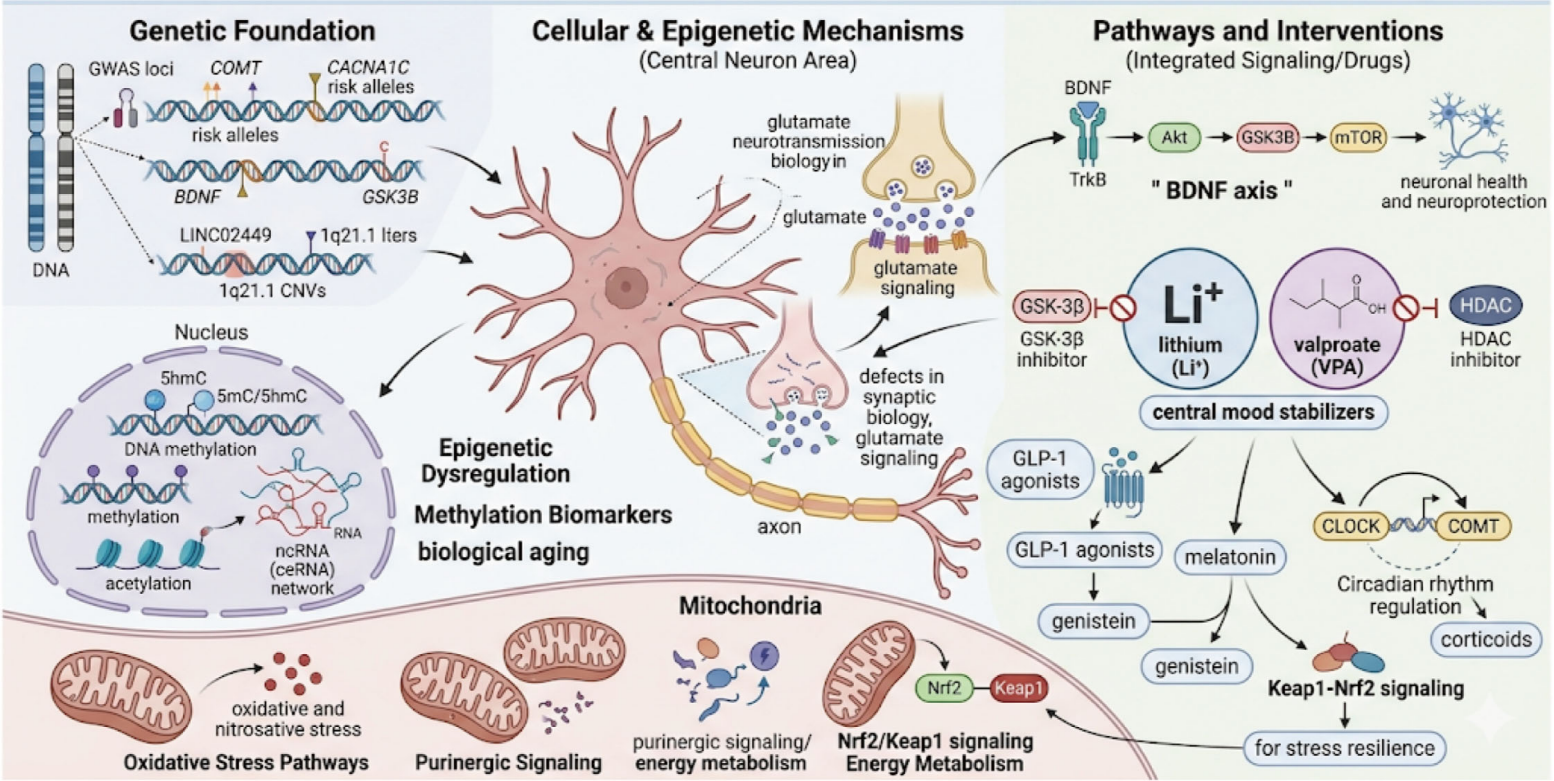
Comprehensive mechanistic pathways of bipolar disorder.

This bibliometric study systematically mapped the development of epigenetics research in bipolar disorder (BD) from 1995 to 2025 on the basis of 1, 154 original English-language articles indexed in WoSCC and Scopus. Overall, the field has shown sustained and accelerating growth, with an annual growth rate of 15.43%. According to the annual publication pattern, the evolution of this field can be divided into three stages: an initial exploration phase (1995–2005), a steady growth phase (2006–2010), and a rapid expansion phase (2011–2025). This temporal pattern indicates that epigenetics has moved from a relatively peripheral topic to a major and increasingly mature research direction in BD. Rather than representing isolated mechanistic studies, the expanding literature reflects the consolidation of a broader interdisciplinary field at the intersection of psychiatry, molecular biology, genetics, and translational neuroscience (**Figure 9**).

### 4.1 Global research landscape and collaboration structure

The country-, institution-, and author-level analyses reveal a research landscape that is both globalized and structurally centralized. The United States occupies the most prominent position in this domain, leading not only in publication output but also in total citations and international collaboration. China has emerged as the second-largest contributor in terms of publication volume, whereas several European countries, particularly France, Germany, and Italy, demonstrate relatively strong international integration as reflected by higher MCP ratios. These findings suggest that BD epigenetics research is driven by a small number of highly influential countries, while newer contributors are still consolidating their global collaborative presence. From a bibliometric perspective, this pattern implies that future influence in the field will depend not only on publication growth, but also on the ability to participate in cross-national research networks and large-scale collaborative studies.

At the institutional level, Harvard University, the University of Texas System, and the University of Toronto represent the most productive affiliations, while the collaboration network highlights Harvard University, the University of Toronto, and the National Institute of Mental Health as major hubs. At the author level, the field is characterized by intense cooperation, as reflected by the very small number of single-author papers, the high mean number of co-authors per document, and the substantial share of internationally co-authored publications. Notably, different bibliometric indicators capture distinct forms of influence: some authors are characterized by sustained productivity and high h-index values, whereas others exert disproportionate academic impact through participation in highly cited, large-scale consortia. Taken together, these findings indicate that knowledge production in BD epigenetics is increasingly team-based, network-driven, and dependent on multi-institutional research infrastructures.

### 4.2 Evolution of knowledge base and paradigm shifts

The timeline analysis of literature co-citations reveals a clear evolutionary logic within the field of bipolar disorder epigenetics. Early foundational research (spanning approximately 2005 to 2010) was predominantly concentrated on exploring basic pharmacological mechanisms. During this phase, critical attention was given to mood stabilizers—specifically, the identification of histone deacetylase (HDAC) inhibitors such as valproic acid (VPA) as key regulators of neural plasticity (18). Pioneering studies with strong citation bursts during this era established a critical molecular nexus: the direct link between HDAC inhibition and the reversal of aberrant chromatin condensation. This conceptual breakthrough formed the core knowledge base, firmly anchoring the disorder’s pathogenesis in epigenetic remodeling (18, 24). Bibliometric clusters from this era highlight the foundational discovery that histone deacetylase (HDAC) inhibitors can reverse maladaptive chromatin states by increasing histone acetylation, thereby promoting a more open chromatin configuration, restoring aberrant gene expression profiles, and ultimately exerting mood-stabilizing and antidepressant-like effects (25–27). Furthermore, studies demonstrating altered expression of histone deacetylases—particularly HDAC2—in patients with bipolar disorder established an early core knowledge base, linking epigenetic enzyme dysregulation to disease pathogenesis (25, 28, 29).

Subsequently, the research paradigm exhibited a gradual transition toward translational investigation and clinically relevant epigenetic applications. During this intermediate phase (approximately 2010–2020), the focus shifted toward exploratory biomarker identification and the integration of epigenetic alterations with clinical phenotypes, particularly emphasizing microRNA dysregulation and patient-state–specific molecular signatures (#3, #4, #6). Rather than establishing definitive diagnostic markers, studies in this period primarily aimed to characterize dynamic and state-dependent epigenetic changes associated with bipolar disorder. This trend is further supported by burst references highlighting large-scale genomic and transcriptomic investigations, which facilitated the linkage between molecular alterations and disease susceptibility (11, 17, 30). In parallel, emerging evidence began to incorporate gene–environment interactions within a broader multi-omics framework, although such genetic components were not the dominant drivers of clustering structure in this stage (31, 32).

Current trends indicate that the field is undergoing a profound transition from “macroscopic disease characterization” to “microscopic precision deconstruction.” Keyword burst analysis demonstrates that since 2020, topics including “autism spectrum disorder, “ “copy number variants (CNV), “ and “differentially expressed genes” have emerged as prominent new research frontiers. This shift reflects a growing recognition within the academic community regarding the shared genetic and epigenetic architecture across different psychiatric diagnoses. Recent high-impact genome-wide association studies (GWAS) and structural variation analyses strongly support this cross-diagnostic trajectory, demonstrating significant polygenic overlap (33) and shared susceptibility loci (such as the 16p11.2 repeat) (34) between bipolar disorder and schizophrenia.

Furthermore, this evolutionary trend highlights ongoing efforts to disentangle the complex pathological mechanisms of bipolar disorder at the gene-specific or single-cell level (5). The research frontier is increasingly focused on how non-coding genetic variants in regulatory regions influence neurodevelopment and synaptic functions (35). Moving forward, the integration of multi-omics data, heavily facilitated by advanced computational algorithms and machine learning, will be essential for translating these complex molecular insights into precision therapeutics (7).

### 4.3 Research hotspots and emerging fronts: systemic physiological networks

Although epigenetic modifications, such as DNA methylation and histone acetylation, remain the core focus of bipolar disorder research, recent keyword burst analyses reveal that the research frontier is rapidly expanding toward systemic physiological networks. Dysfunctions in the hypothalamic-pituitary-adrenal (HPA) axis, circadian rhythm disruptions, and oxidative stress are no longer viewed as isolated pathological events; rather, they are intricately intertwined through complex epigenetic regulation.

Within this network, recent literature highlights the HPA axis as a critical node linking stress responses to emotional dysregulation (36). Bibliometric evidence points to a growing intersection between neuroendocrine dysfunction and genetic vulnerabilities; for instance, variations in the CACNA1C gene (37) and the use of GR-dominant corticosteroids (38) have been frequently cited as exacerbators of HPA axis dysregulation and psychiatric instability.

Parallel to neuroendocrine research, chronobiology has emerged as a major thematic cluster. Studies consistently link circadian and social rhythm disruptions to BD pathogenesis, emphasizing that genetic predispositions (such as GSK3-ß and COMT genotypes) intricately modulate patient responses to mood stabilizers like valproate (39). Furthermore, cross-domain literature reveals that these physiological systems are highly interactive. For example, hypomelatoninaemia in BD is increasingly recognized for its role in exacerbating oxidative stress, thereby bridging circadian dysregulation with redox imbalance and neuroinflammation (40).

Consequently, oxidative stress has surfaced as a prominent research hotspot. The focus of recent publications has deepened into specific molecular pathways, such as the downregulation of the NFE2L2 (Nrf2) antioxidant signaling pathway (41) and reduced SOD1 levels (42). Crucially, current studies heavily emphasize the cross-talk between oxidative stress and neuroplasticity. Evidence shows that stress-induced redox imbalance via mitochondrial dysfunction (43) negatively impacts brain-derived neurotrophic factor (BDNF) expression, thereby driving mood cycling and cognitive deficits (44).

This bibliometric shift toward interconnected physiological networks holds profound translational implications. The literature is pivoting from single-target drugs toward multi-targeted therapeutic strategies aimed at correcting these systemic imbalances. Emerging research exploring GLP-1 receptor agonists for HPA regulation (45), melatonin for circadian restoration (46), and antioxidant-modulating agents (such as genistein (47) and lithium (48) reflects the field’s overarching trajectory toward precision medicine and comprehensive, system-level management of bipolar disorder.

### 4.4 Implications and methodological boundaries

Beyond tracing historical trajectories, our mapping of BD epigenetics reveals a research landscape undergoing profound structural changes. The data highlight a distinct institutional and geographical clustering, indicating that the intellectual core of this field remains heavily concentrated within a few prominent hubs. However, while the key players are concentrated, the scientific focus is rapidly expanding outward. The surging prominence of keywords related to machine learning, GWAS, and biomarker discovery signals a definitive pivot away from pure mechanism exploration towards translational applications. This shift underscores an urgent clinical demand for biologically informed psychiatric classification and precision treatment strategies. To sustain this momentum, future breakthroughs will increasingly depend on massive, cross-disciplinary collaborations. Moving forward, isolated studies are likely to yield diminishing returns compared to multi-center consortia capable of standardizing phenotypes and validating findings across diverse, global cohorts.

While these macroscopic trends offer valuable insights, they must be contextualized within the inherent boundaries of bibliometric methodology. By restricting our retrieval to English-language literature in the WoSCC and Scopus databases, we inevitably introduce an element of linguistic and regional selection bias, potentially underrepresenting valuable findings published in local or specialized psychiatric journals. Furthermore, our reliance on citation metrics means the analysis is subject to a natural time lag. Consequently, highly innovative or paradigm-shifting papers published within the last two years might not yet register as central nodes in our co-citation networks simply because they have not had sufficient time to accumulate citations. Most importantly, it is crucial to recognize that bibliometric networks map the flow of scientific attention and collaborative productivity, rather than directly assessing the methodological rigor or clinical efficacy of individual experiments. Thus, our findings serve as a macroscopic navigational tool for the evolution of BD epigenetics, complementing rather than replacing the granular quality assessment provided by traditional systematic reviews.

## Data Availability

The original contributions presented in the study are included in the article/**Supplementary Material**. Further inquiries can be directed to the corresponding author.
