# Supplementary material for: Epigenetics of bipolar disorder: a bibliometric landscape and visualization analysis
Source: Front Psychiatry. 2026 Jun 5;17:1804155. doi: 10.3389/fpsyt.2026.1804155 (PMC13279425; doi:10.3389/fpsyt.2026.1804155)
Supplement: Supplementary file 1 [file Table1.docx]

**Title Page**

**Epigenetics of Bipolar Disorder: A Bibliometric Landscape and Visualization Analysis**

**Supplementary Tables:**

**Tabel S1** Top 10 Authors of epigenetic research publications for Bipolar Disorder

**Tabel S2.** Top 15 Journals by Influence Rank According to Bibliometrix Analysis

**Table S3.** Top 10 Most Cited Publications Based on Bibliometrix Analysis.

**Table S4.** Bibliometric Summary of epigenetic research publications for Bipolar Disorder.

**Table S5.** Most Relevant Countries by Corresponding Author Contributions.

**Table S6.** TOP 10 Affiliations and Article Counts of epigenetic research publications for Bipolar Disorder.

**Table S1:** Top 10 Authors of epigenetic research publications for Bipolar Disorder (1995–2025)

| **Rank** | **Author** | **h_index** | **g_index** | **m_index** | **TC** | **NP** | **PY_start** | **Articles** | **Articles Fractionalized** |
| --- | --- | --- | --- | --- | --- | --- | --- | --- | --- |
| 1 | KATO T | 16 | 25 | 0.533 | 1264 | 25 | 1996 | 25 | 5.00 |
| 2 | QUEVEDO J | 15 | 21 | 1 | 738 | 21 | 2011 | 21 | 2.31 |
| 3 | GRAYSON DR | 14 | 15 | 0.667 | 1449 | 15 | 2005 | 15 | 3.47 |
| 4 | VALVASSORI SS | 14 | 18 | 0.933 | 734 | 18 | 2011 | 18 | 2.09 |
| 5 | BELLIVIER F | 13 | 18 | 0.448 | 2407 | 18 | 1997 | 18 | 1.68 |
| 6 | FRIES GR | 13 | 19 | 0.867 | 585 | 19 | 2011 | 19 | 2.22 |
| 7 | IWAMOTO K | 13 | 16 | 0.565 | 869 | 16 | 2003 | 16 | 2.11 |
| 8 | COLLIER DA | 12 | 12 | 0.414 | 2887 | 12 | 1997 | 12 | 1.21 |
| 9 | LEBOYER M | 12 | 16 | 0.414 | 2325 | 16 | 1997 | 16 | 1.16 |
| 10 | NÖTHEN MM | 12 | 16 | 0.48 | 4230 | 16 | 2001 | 16 | 0.36 |

**Table S2:** Top 15 Journals by Influence Rank According to Bibliometrix Analysis(1995–2025)

| **Rank** | **Source** | **h_index** | **g_index** | **m_index** | **TC** | **NP** | **PY_start** | **Articles** | **JCR** | **IF** |
| --- | --- | --- | --- | --- | --- | --- | --- | --- | --- | --- |
| 1 | MOLECULAR PSYCHIATRY | 27 | 42 | 0.964 | 3300 | 42 | 1998 | 42 | Q1 | 10.1 |
| 2 | TRANSLATIONAL PSYCHIATRY | 23 | 41 | 1.533 | 1734 | 42 | 2011 | 42 | Q1 | 6.2 |
| 3 | SCHIZOPHRENIA RESEARCH | 19 | 30 | 0.864 | 1596 | 30 | 2004 | 30 | Q1 | 3.5 |
| 4 | JOURNAL OF PSYCHIATRIC RESEARCH | 17 | 25 | 1 | 1279 | 25 | 2009 | 25 | Q2 | 3.2 |
| 5 | JOURNAL OF AFFECTIVE DISORDERS | 17 | 30 | 0.607 | 962 | 36 | 1998 | 1 | Q1 | 4.9 |
| 6 | PLOS ONE | 16 | 23 | 0.941 | 1508 | 23 | 2009 | 23 | Q2 | 2.6 |
| 7 | BIPOLAR DISORDERS | 16 | 22 | 0.667 | 676 | 22 | 2002 | 22 | Q1 | 4.5 |
| 8 | AMERICAN JOURNAL OF MEDICAL GENETICS PART B-NEUROPSYCHIATRIC GENETICS | 14 | 17 | 0.609 | 1204 | 17 | 2003 | 17 | Q3 | 1.5 |
| 9 | NEUROSCIENCE LETTERS | 12 | 14 | 0.522 | 539 | 14 | 2003 | 14 | Q4 | 2.0 |
| 10 | NEUROPSYCHOPHARMACOLOGY | 11 | 11 | 0.423 | 1039 | 11 | 2000 | 11 | Q1 | 7.1 |
| 11 | INTERNATIONAL JOURNAL OF NEUROPSYCHOPHARMACOLOGY | 11 | 11 | 0.407 | 710 | 11 | 1999 | 11 | Q1 | 3.7 |
| 12 | AMERICAN JOURNAL OF MEDICAL GENETICS, PART B: NEUROPSYCHIATRIC GENETICS | 11 | 17 | 0.458 | 538 | 17 | 2002 | 17 | Q3 | 1.5 |
| 13 | BIOLOGICAL PSYCHIATRY | 10 | 10 | 0.333 | 754 | 10 | 1996 | 10 | Q1 | 9.0 |
| 14 | PROCEEDINGS OF THE NATIONAL ACADEMY OF SCIENCES OF THE UNITED STATES OF AMERICA | 9 | 9 | 0.429 | 2152 | 9 | 2005 | 9 | Q1 | 9.1 |
| 15 | FRONTIERS IN PSYCHIATRY | 9 | 15 | 0.9 | 226 | 20 | 2016 | 20 | Q2 | 3.2 |

**Table S3:** Top 10 Most Cited Publications Based on Bibliometrix Analysis(1995–2025)

| **Rank** | **author** | **title** | **journal** | **years** | **DOI** | **Total Citations** | **TC per Year** | **Normalized TC** |
| --- | --- | --- | --- | --- | --- | --- | --- | --- |
| 1 | LOHMUELLER KE | Meta-analysis of genetic association studies supports a contribution of common variants to susceptibility to common disease | NAT GENET | 2003 | 10.1038/ng1071 | 1672 | 72.70 | 6.01 |
| 2 | Schizophrenia Psychiatric Genome-Wide Association Study (GWAS) Consortium | Genome-wide association study identifies five new schizophrenia loci | NAT GENET | 2011 | 10.1038/ng.940 | 1528 | 101.87 | 14.04 |
| 3 | PHIEL CJ | Histone deacetylase is a direct target of valproic acid, a potent anticonvulsant, mood stabilizer, and teratogen | J BIOL CHEM | 2001 | 10.1074/jbc.M101287200 | 1466 | 58.64 | 5.20 |
| 4 | STAHL EA | Genome-wide association study identifies 30 loci associated with bipolar disorder | NAT GENET | 2019 | 10.1038/s41588-019-0397-8 | 1066 | 152.29 | 19.90 |
| 5 | Michael Rutter | Continuities and discontinuities in psychopathology between childhood and adult life | J CHILD PSYCHOL PSYC | 2006 | 10.1111/j.1469-7610.2006.01614.x | 639 | 31.95 | 5.49 |
| 6 | MILL J | Epigenomic profiling reveals DNA-methylation changes associated with major psychosis | AM J HUM GENET | 2008 | 10.1016/j.ajhg.2008.01.008 | 609 | 33.83 | 6.85 |
| 7 | Network and Pathway Analysis Subgroup of Psychiatric Genomics Consortium | Psychiatric genome-wide association study analyses implicate neuronal, immune and histone pathways | NAT NEUROSCI | 2015 | 10.1038/nn.3922 | 582 | 52.91 | 12.23 |
| 8 | WILLIAMS RSB | A common mechanism of action for three mood-stabilizing drugs | NATURE | 2002 | 10.1038/417292a | 579 | 24.13 | 3.20 |
| 9 | CALKINS KL | Fetal origins of adult disease | CURR PROB PEDIATR AD | 2011 | 10.1016/j.cppeds.2011.01.001 | 505 | 33.67 | 4.64 |
| 10 | GRAYSON DR | Reelin promoter hypermethylation in schizophrenia | P NATL ACAD SCI USA | 2005 | 10.1073/pnas.0503736102 | 496 | 23.62 | 4.18 |

**Table S4** Bibliometric Summary of epigenetic research publications for Bipolar Disorder (1995–2025)

| **Description** | **Results** |
| --- | --- |
| MAIN INFORMATION ABOUT DATA |  |
| Timespan | 1995:2025 |
| Sources (Journals, Books, etc) | 407 |
| Documents | 1154 |
| Annual Growth Rate % | 15.43 |
| Document Average Age | 8.89 |
| Average citations per doc | 48.54 |
| References | 31114 |
| DOCUMENT CONTENTS |  |
| Keywords Plus (ID) | 8704 |
| Author's Keywords (DE) | 2476 |
| AUTHORS |  |
| Authors | 7220 |
| Authors of single-authored docs | 47 |
| AUTHORS COLLABORATION |  |
| Single-authored docs | 50 |
| Co-Authors per Doc | 9.66 |
| International co-authorships % | 18.37 |
| DOCUMENT TYPES |  |
| article | 1154 |

**Table S5.** Most Relevant Countries by Corresponding Author Contributions

| **Rank** | **Country** | **Articles** | **Articles %** | **SCP** | **MCP** | **MCP %** | **TC** | **Average Article Citations** | |
| --- | --- | --- | --- | --- | --- | --- | --- | --- | --- |
| 1 | USA | 191 | 16.6 | 129 | 62 | 32.5 | 16181 | 84.70 |  |
| 2 | CHINA | 74 | 6.4 | 59 | 15 | 20.3 | 1355 | 18.30 |  |
| 3 | JAPAN | 35 | 3 | 30 | 5 | 14.3 | 1520 | 43.40 |  |
| 4 | CANADA | 33 | 2.9 | 18 | 15 | 45.5 | 1402 | 42.50 |  |
| 5 | UNITED KINGDOM | 31 | 2.7 | 21 | 10 | 32.3 | 1854 | 59.80 |  |
| 6 | GERMANY | 26 | 2.3 | 10 | 16 | 61.5 | 1419 | 54.60 |  |
| 7 | ITALY | 25 | 2.2 | 15 | 10 | 40 | 1022 | 40.90 |  |
| 8 | BRAZIL | 21 | 1.8 | 9 | 12 | 57.1 | 649 | 30.90 |  |
| 9 | FRANCE | 19 | 1.6 | 6 | 13 | 68.4 | 403 | 21.20 |  |
| 10 | IRAN | 17 | 1.5 | 15 | 2 | 11.8 | 151 | 8.90 |  |

Abbreviation: SCP :Single Country Publications; MCP :Multiple Country Publications; TC: Total Citation

**Table S6**. TOP 10 Affiliations and Article Counts (1995–2025)

| **Affiliation** | **Articles** |
| --- | --- |
| HARVARD UNIVERSITY | 120 |
| UNIVERSITY OF TEXAS SYSTEM | 69 |
| UNIVERSITY OF TORONTO | 68 |
| UNIVERSITY OF CALIFORNIA SYSTEM | 62 |
| ICAHN SCHOOL OF MEDICINE AT MOUNT SINAI | 61 |
| HARVARD UNIVERSITY MEDICAL AFFILIATES | 60 |
| HARVARD MEDICAL SCHOOL | 58 |
| UNIVERSITY OF LONDON | 58 |
| BOSTON UNIVERSITY | 52 |
| UNIVERSITY OF BARCELONA | 49 |
